# Trends in All-Cause Mortality across Gestational Age in Days for Children Born at Term

**DOI:** 10.1371/journal.pone.0144754

**Published:** 2015-12-14

**Authors:** Chun Sen Wu, Yuelian Sun, Ellen Aagaard Nohr, Jørn Olsen

**Affiliations:** 1 Research Unit on Gynecology and Obstetrics, Institute of Clinical Research, University of Southern Denmark, Odense, Denmark; 2 Department of Gynecology and Obstetrics, Odense University Hospital, Odense, Denmark; 3 Section for Epidemiology, Department of Public Health, Aarhus University, Aarhus, Denmark; 4 Department of Epidemiology, School of Public Health, University of California Los Angeles, Los Angeles, CA, United States of America; University of Helsinki, FINLAND

## Abstract

**Background:**

Term birth is a gestational age from 259 days to 293 days. However trends in mortality according to gestational ages in days have not yet been described in this time period.

**Methods and Findings:**

Based on nation-wide registries, we conducted a population-based cohort study among all children born at term in Denmark from 1997 to 2004 to estimate differences in mortality across gestational ages in days among singletons born at term. We studied early-neonatal mortality, neonatal mortality, infant mortality, and five-year mortality. Children were followed from birth up to the last day of the defined mortality period or December 31, 2009. A total of 360,375 singletons born between 259 and 293 days of gestation were included in the study. Mortality decreased with increasing gestational age in days and the highest mortality was observed among children born at 37 week of gestation. A similar pattern was observed when analyses were restricted to children born to by mothers without pregnancy complications.

**Conclusions:**

This study demonstrates heterogeneity in mortality rates even among singletons born at term. The highest mortality was observed among children born 37 weeks of gestation, which call for cautions when inducing labor in term pregnancies just reaching 37 weeks of gestation. The findings support that 37 weeks of gestation should be defined as early term.

## Introduction

The International Classification of Diseases defines a term pregnancy as a delivery between 259 days (37 weeks 0 days) and 293 days (41 weeks 6 days).[1] Increasing evidence indicates that births between 37 weeks 0 days and 38 weeks 6 days are associated with an increased mortality and morbidity[2–6] and redefining “term pregnancy” has therefore been proposed,[5,7,8] for example to classify early-term birth as delivery between 37 weeks 0 day and 38 weeks 6 days, full-term birth as delivery between 39 weeks 0 day through 40 weeks 6 days, and late-term birth as delivery between 41 weeks 0 day through 41 weeks 6 days.[9] To our best knowledge that all the evidences were based on completed gestational week. However, fetal growth and development is rapid and using completed gestational week may be too crude. Little is known about mortality across gestational age measured in days among children born at term.

We conducted a descriptive population-based cohort study to examine trends in all-cause mortality including *early-neonatal mortality*, *neonatal mortality*, *infant mortality*, and *five-year mortality* according to gestational age measured in days among children born at term (from day 259 through day 293 of gestation). We took maternal morbidity during pregnancy, stillbirth, and congenital malformation into consideration.

## Materials and Methods

### Ethic Statement

According to Danish law, register-based studies do not require consent from individuals when personal identifiers are encrypted and stored by a trusted third party (Statistic Denmark). This study was approved by the Danish Data Protection Agency (J.nr.2008-41-2680).

### Study population

We identified live-born singletons of women giving birth from January 1 1997 to December 31 2004 in Denmark (N = 504,519) according to the Danish Medical Birth Register (DMBR) which includes all births in Denmark since 1973.[10] DMBR holds information on maternal age, maternal parity at birth, gestational age, and birth weight.[10] All live-born children and new residents in Denmark are assigned a unique civil registration number, which we used to link information from several national registries at the individual level.

### Gestational age

Information on gestational age was reported in days by the midwife attending the delivery using a mandatory coding sheet.[11] Antenatal care is tax paid in Denmark and ultrasound measurements have been widely used to determine gestational age in nearly all pregnancies since 1995.[12,13] Gestational age was measured based on either early biparietal diameter (BPD) measurement or detailed information on the woman’s last menstrual period (LMP).[14] A discrepancy may exist between gestational age estimated by LMP and BPD [15,16] and a difference between the two methods exceeding 1 or 2 weeks, gestational age estimated from LMP will be “corrected” according to local ultrasound guidelines.[14]

### Outcomes: mortality

We explored both short-term and longer-term all-cause mortality rates. *Early-neonatal mortality* was any death up to 7 days after birth, *neonatal mortality* was any death up to 28 days after birth, *infant mortality* was any death up to 365 days after birth, and *five-year mortality* was any death up to 1825 days after birth.

### Covariates

Information on caesarean section was based on the Danish version of the 10^th^ revision (ICD-10: O82) obtained from the National Hospital Register that holds nationwide data on all admissions to any Danish hospital since 1977 and on all outpatient visits since 1995.[17] Data on maternal education and marital status at the time of birth from 1997 through 2004 were obtained from Statistics Denmark. Missing values on maternal education were replaced by available information in the preceding or following five years year. Missing values on marital status were replaced by available information in the preceding or following three years, whichever came first.

### Congenital malformations and stillbirth

Information on any congenital malformation obtained from the Danish Hospital Register using ICD-10: Q00 –Q99. Information on stillbirth obtained from the Denmark statistics and the Danish Hospital register.

### Maternal morbidity

We identified all hospital records within one year before delivery or within 30 days after delivery except for hospital visit due to prenatal care (ICD10 codes: Z00 –Z99) and delivery (ICD10 codes: O80 –O84).

### Statistical Analyses

We used Cox regression model to estimate hazard ratios (HRs) with 95% confidence interval (95% CI). Children were followed from birth, until death, emigration, the end day of the defined mortality period (7, 28, 365, or 1825 days after birth), or December 31 2009, whichever came first to estimate hazard ratios for *earl-neonatal mortality*, *neonatal mortality*, *infant mortality*, and *five-year mortality*, respectively. Statistical analyses were performed using STATA version 11 (Stata Corp., College Station, Texas, USA).

Mortality by gestational age in days was modelled by using restricted cubic splines with four knots (266 days, 273 days, 280 days, and 287 days) and using 280 days of gestational age as the reference. We performed crude, adjusted, and restricted analyses. In Model 1, results were adjusted for caesarean section (yes or no), maternal age (<25, 25–30, 30–35, > = 35), parity (1, 2, and 3), and birth year (1997–1999, 2000–2002, 2003–2005, and 2006–2008). In Model 2, the results were additionally adjusted for maternal education (low, middle, and high) and maternal cohabitant status during birth (yes, no). Afterwards, we restricted our analyses to pregnancies without any registered morbidity within one year before delivery or within 30 days after delivery using the same adjustments as in Model 2. Finally, we restricted our analyses to children without any congenital malformations.

### Sensitivity analyses

In order to know how sensitive when gestational days are grouped as gestational week, we repeated the above analyses by categorizing gestational age in days into week 37 (between 259 and 265 days), week 38 (between 266 and 272 days), week 39 (between 273 and 279 days), week 40 (between 280 and 286 days), and week 41 (between 287 and 293 days). We also explored the distribution of the stillbirth according gestational age in days.

## Results

**Fig 1:**
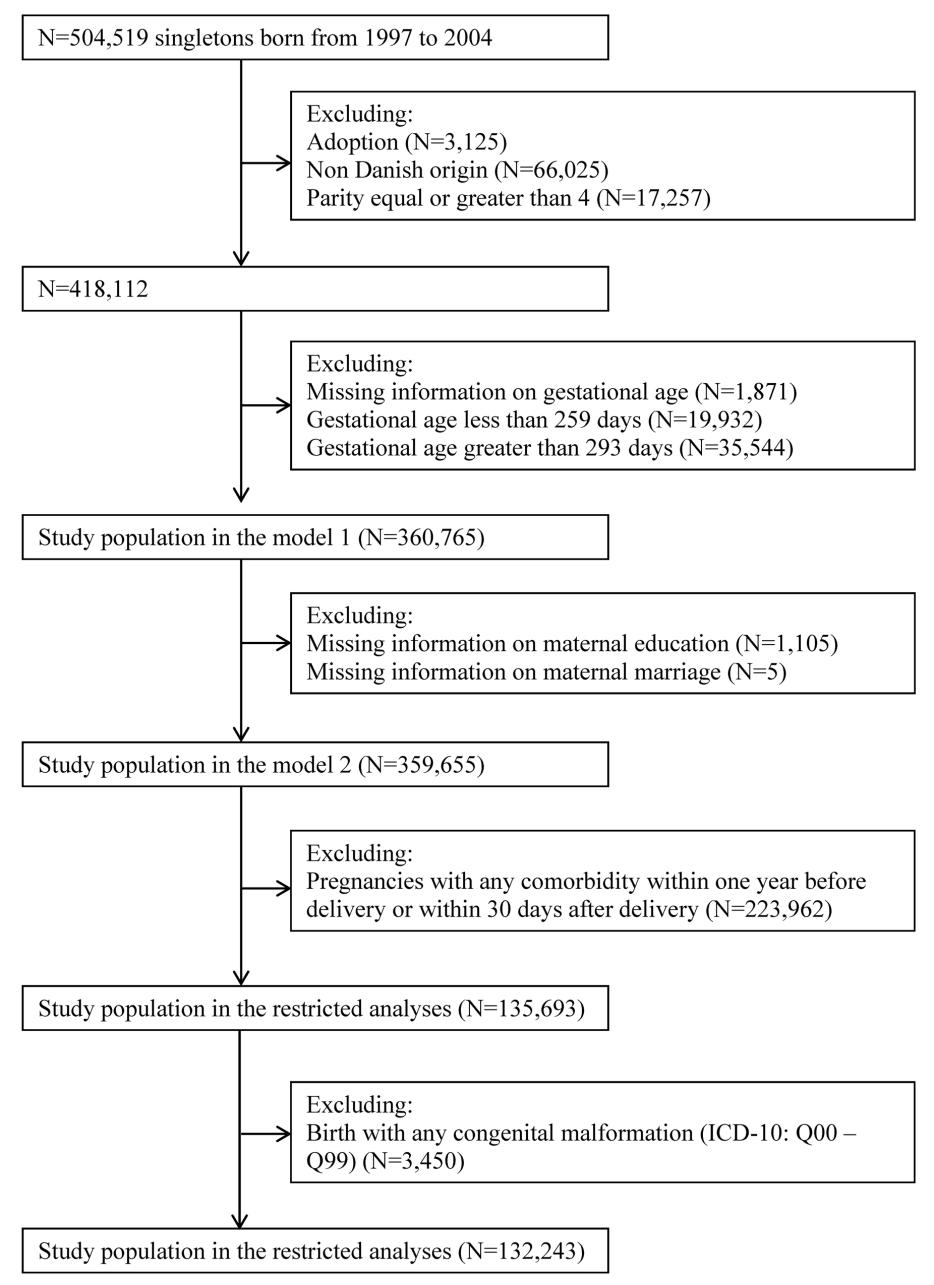
Study population.

Among live-born singletons born between January 1 1997 and December 31 2004 in Denmark (N = 504,519), we excluded children who were adopted (n = 3,512), singletons whose mothers were not of Danish origin (N = 66,025), maternal parity at birth larger than three (N = 17,257), missing information on gestational age (N = 1,871), gestational age less than 259 days (N = 19,932) or larger than 293 days (N = 35,544) leaving 360,765 singletons in the study population. We also generated a subset of the study population with 359,655 singletons after excluding singletons with missing information on maternal education (N = 1,105) and maternal cohabitant status (N = 5) (Fig 1).

A total of 360,375 singletons were born between 259 and 293 days of gestation from 1997 to 2004. The proportion of early term birth (between 37 weeks 0 days and 38 weeks 6 days) increased with birth year while late term birth (between 41 week 0 and 7 days) decreased. The proportion of caesarean section was higher among children born early-term (Table 1).

**Table 1:** Characteristics of the study population according to completed gestational week.

	Competed gestational week									
	Week 37		Week 38		Week 39		Week 40		Week 41	
	No.	%	No.	%	No.	%	No.	%	No.	%
Gender										
Boy	9,923	53·14	25,315	52·08	45,733	50·69	60,415	50·18	41,943	50·62
Girl	8,750	46·86	23,296	47·92	44,496	49·31	59,979	49·82	40,915	49·38
Caesarean										
No	13,904	74·46	34,073	70·09	76,873	85·20	110,009	91·37	74,104	89·43
Yes	4,769	25·54	14,538	29·91	13,356	14·80	10,385	8·63	8,754	10·57
Parity										
1	8,926	47·80	20,266	41·69	38,898	43·11	52,478	43·59	38,467	46·43
2	6,939	37·16	20,301	41·76	38,101	42·23	50,220	41·71	32,734	39·51
3	2,808	15·04	8,044	16·55	13,230	14·66	17,696	14·70	11,657	14·07
Maternal age										
<25	2,640	14·14	6,154	12·66	11,924	13·22	15,677	13·02	10,700	12·91
25-	6,950	37·22	17,309	35·61	33,901	37·57	46,037	38·24	31,719	38·28
30-	6,310	33·79	17,391	35·78	31,995	35·46	42,995	35·71	29,629	35·76
35-	2,773	14·85	7,757	15·96	12,409	13·75	15,685	13·03	10,810	13·05
Birth year										
1997	2,020	10·82	5,435	11·18	11,391	12·62	16,674	13·85	11,113	13·41
1998	2,134	11·43	5,416	11·14	10,882	12·06	15,893	13·20	11,052	13·34
1999	2,124	11·37	5,461	11·23	11,099	12·30	15,417	12·81	10,941	13·20
2000	2,371	12·70	5,980	12·30	11,845	13·13	15,262	12·68	10,566	12·75
2001	2,294	12·29	5,953	12·25	11,057	12·25	14,631	12·15	10,298	12·43
2002	2,423	12·98	6,241	12·84	11,058	12·26	14,118	11·73	9,911	11·96
2003	2,571	13·77	6,779	13·95	11,290	12·51	14,191	11·79	9,787	11·81
2004	2,736	14·65	7,346	15·11	11,607	12·86	14,208	11·80	9,190	11·09
Maternal education										
Low	4,505	24·13	10,998	22·62	19,718	21·85	25,998	21·59	17,575	21·21
Middle	7,239	38·77	18,744	38·56	34,590	38·34	45,472	37·77	30,835	37·21
High	6,848	36·67	18,722	38·51	35,632	39·49	48,574	40·35	34,210	41·29
Missing	81	0·43	147	0·30	289	0·32	350	0·29	238	0·29
Marriage										
Others	8,915	47·74	21,694	44·63	40,687	45·09	54,057	44·90	37,562	45·33
Married	9,758	52·26	26,916	55·37	49,541	54·91	66,334	55·10	45,295	54·67
Missing	0	0·00	1	0·00	1	0·00	3	0·00	1	0·00
Comorbidity										
No	5,842	31·29	16,863	34·69	35,046	38·84	47,324	39·31	30,993	37·40
Yes	12,831	68·71	31,748	65·31	55,183	61·16	73,070	60·69	51,865	62·60
Stillbirth										
Yes	131		127		128		179		155	
Missing	1		2		2		3		3	

**Fig 2:**
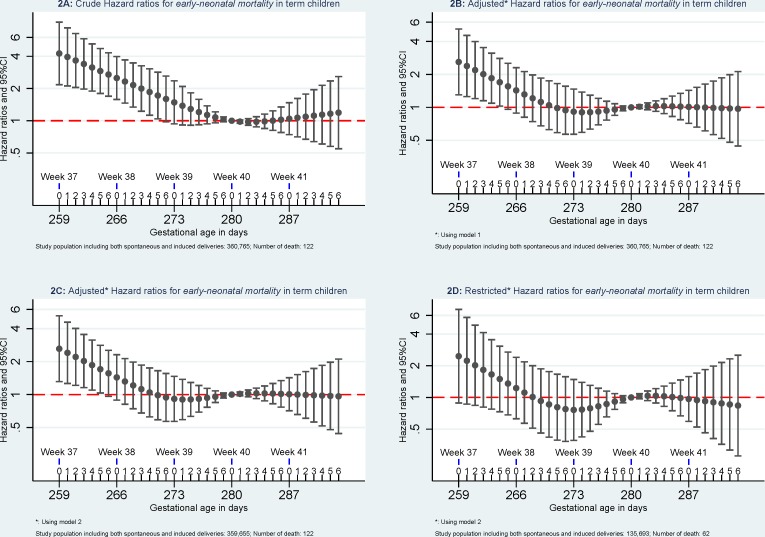
Hazard ratios for early neonatal mortality. Model 1: the results were adjusted for caesarean section (yes or no), maternal age (<25, 25–30, 30–35, > = 35), parity (1, 2, and 3), and birth year (1997–1999, 2000–2002, 2003–2005, and 2006–2008). Model 2, the results were additionally adjusted for maternal education (low, middle, and high) and maternal cohabitant status during birth (yes, no). Restricted analyses: the analyses were restricted to pregnancies without registered morbidity within one year before delivery or within 30 days after delivery using the same adjustments in Model 2.

**Fig 3:**
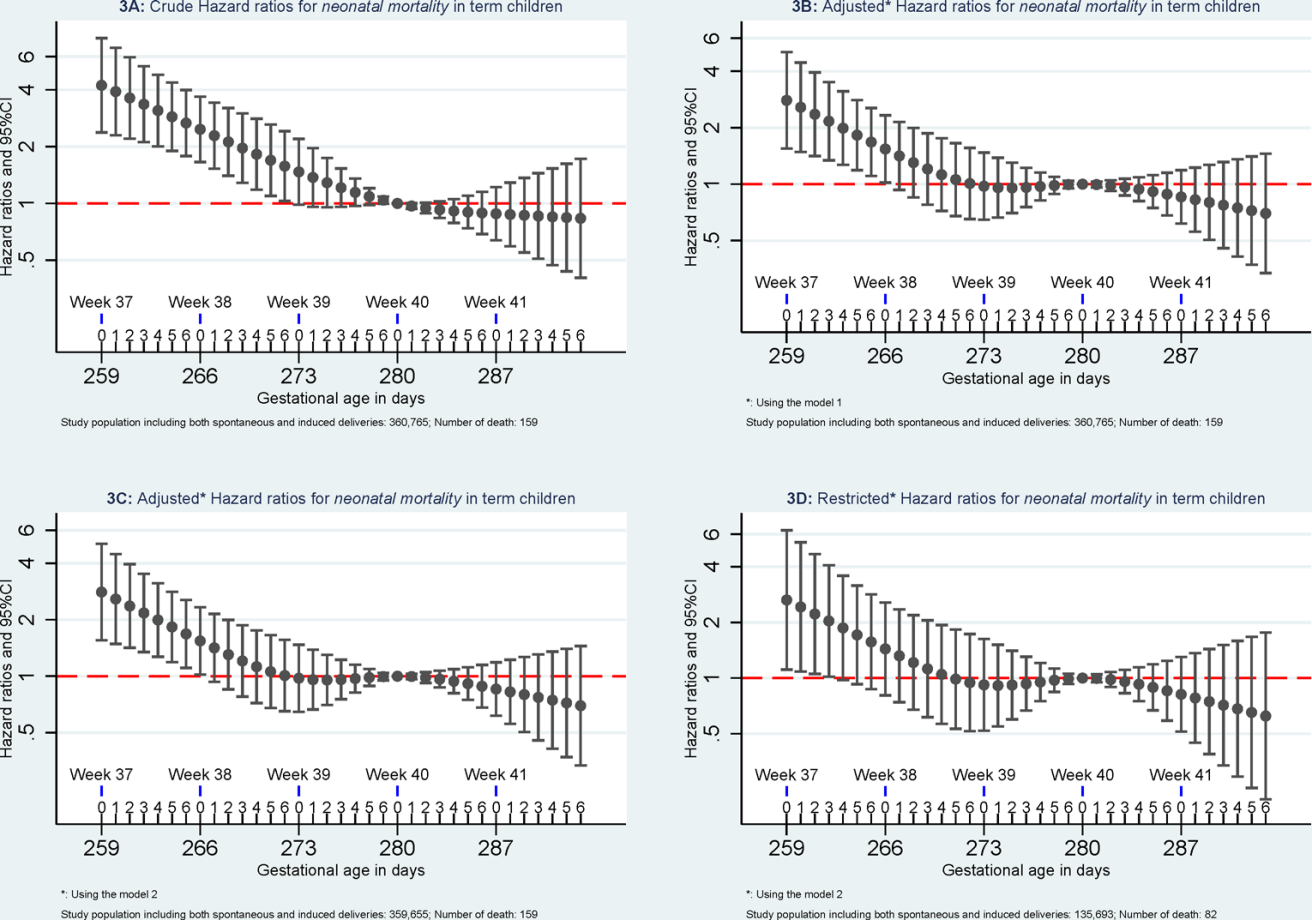
Hazard ratios for neonatal mortality. Model 1: the results were adjusted for caesarean section (yes or no), maternal age (<25, 25–30, 30–35, > = 35), parity (1, 2, and 3), and birth year (1997–1999, 2000–2002, 2003–2005, and 2006–2008). Model 2, the results were additionally adjusted for maternal education (low, middle, and high) and maternal cohabitant status during birth (yes, no). Restricted analyses: the analyses were restricted to pregnancies without registered morbidity within one year before delivery or within 30 days after delivery using the same adjustments in Model 2.

**Fig 4:**
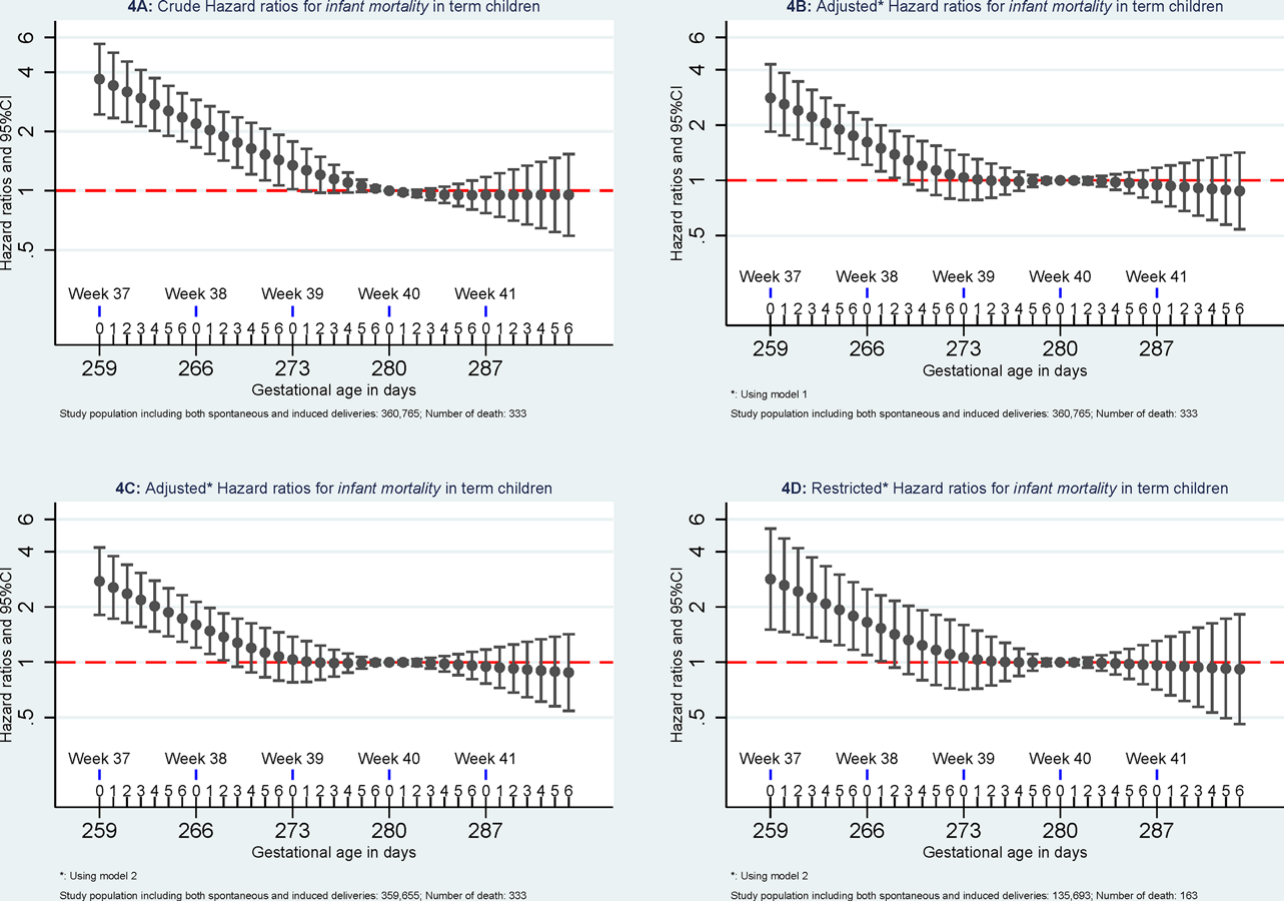
Hazard ratios for infant mortality. Model 1: the results were adjusted for caesarean section (yes or no), maternal age (<25, 25–30, 30–35, > = 35), parity (1, 2, and 3), and birth year (1997–1999, 2000–2002, 2003–2005, and 2006–2008). Model 2, the results were additionally adjusted for maternal education (low, middle, and high) and maternal cohabitant status during birth (yes, no). Restricted analyses: the analyses were restricted to pregnancies without registered morbidity within one year before delivery or within 30 days after delivery using the same adjustments in Model 2.

**Fig 5:**
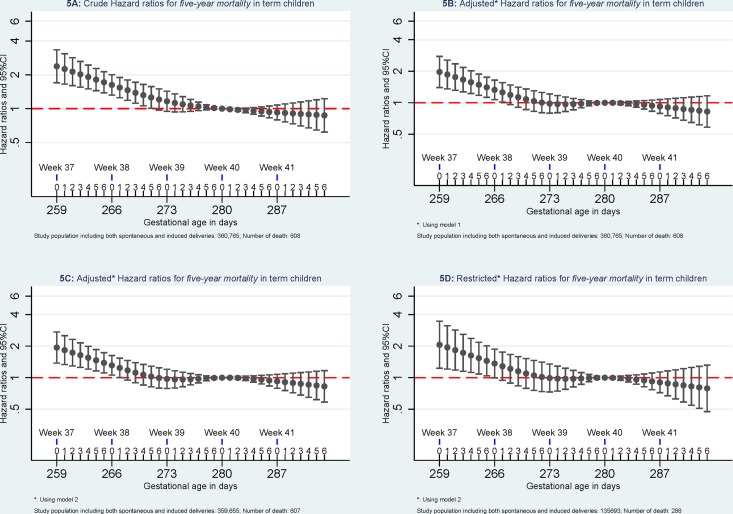
Hazard ratios for five-year mortality. Model 1: the results were adjusted for caesarean section (yes or no), maternal age (<25, 25–30, 30–35, > = 35), parity (1, 2, and 3), and birth year (1997–1999, 2000–2002, 2003–2005, and 2006–2008). Model 2, the results were additionally adjusted for maternal education (low, middle, and high) and maternal cohabitant status during birth (yes, no). Restricted analyses: the analyses were restricted to pregnancies without registered morbidity within one year before delivery or within 30 days after delivery using the same adjustments in Model 2.

Compared to *mortality* of singletons born at 280 days of gestational age, the highest *mortality* was found among singletons born at week 37 of gestational age. *Mortality* decreased with increasing gestational age in days until 280 days of gestation. (Figs 2–5).

**Fig 6:**
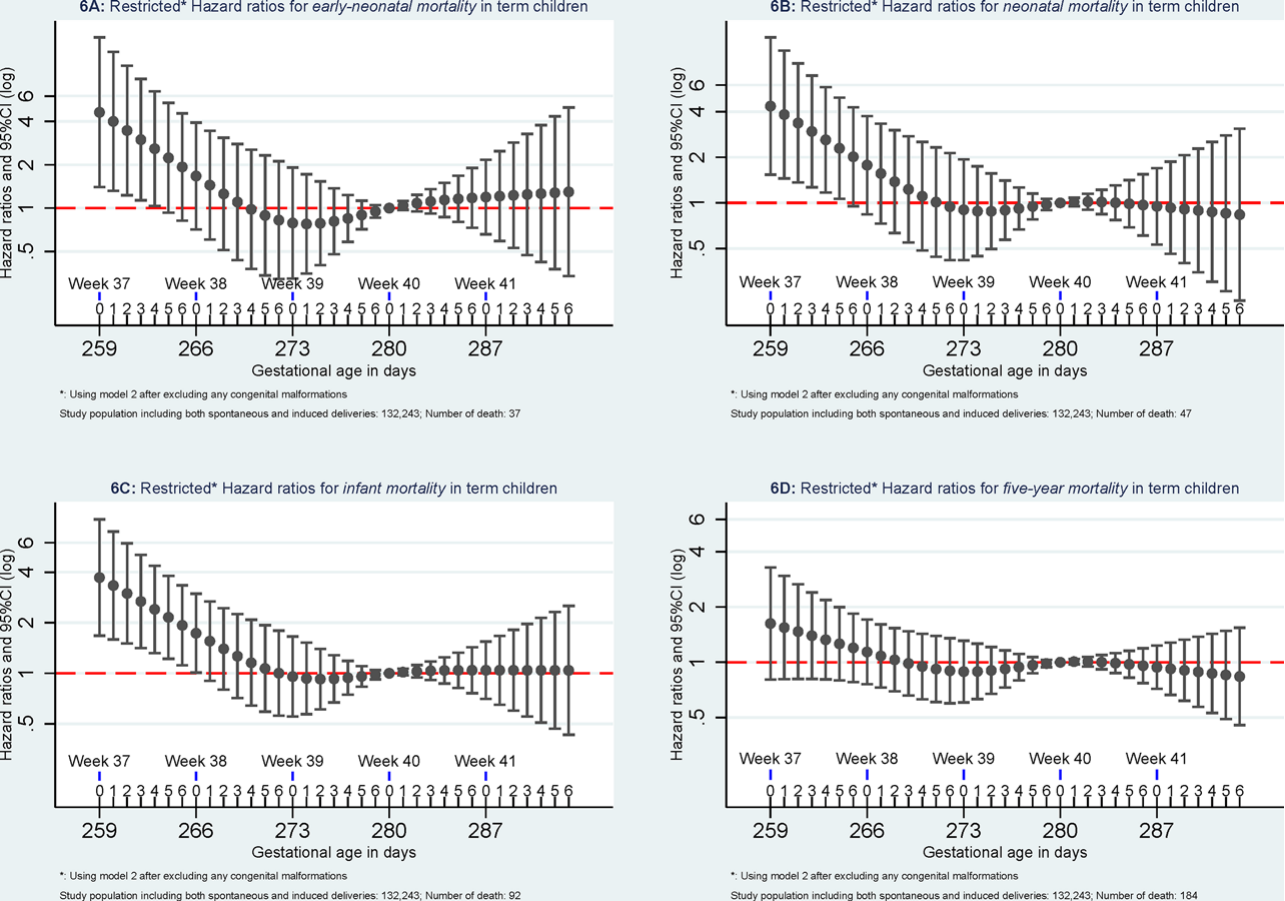
Mortality after excluding pregnancies with any maternal diseases and children with any congenital malformations. The analyses were done using Model 2 and excluding pregnancies without registered morbidity within one year before delivery or within 30 days after delivery and excluding children with any registered congenital malformations.

When excluding pregnancies with any morbidity within one year before delivery or within 30 days after delivery, *early-neonatal mortality* and *neonatal mortality* attenuated and were no longer significant but similar patterns were observed for *infant mortality and five-year mortality*. When further excluding children with congenital malformation, mortality at 37 of gestation remained significant higher for *early-neonatal mortality*, *neonatal mortality*, and *infant mortality* but not for *five-year mortality* (Fig 6).

**Fig 7:**
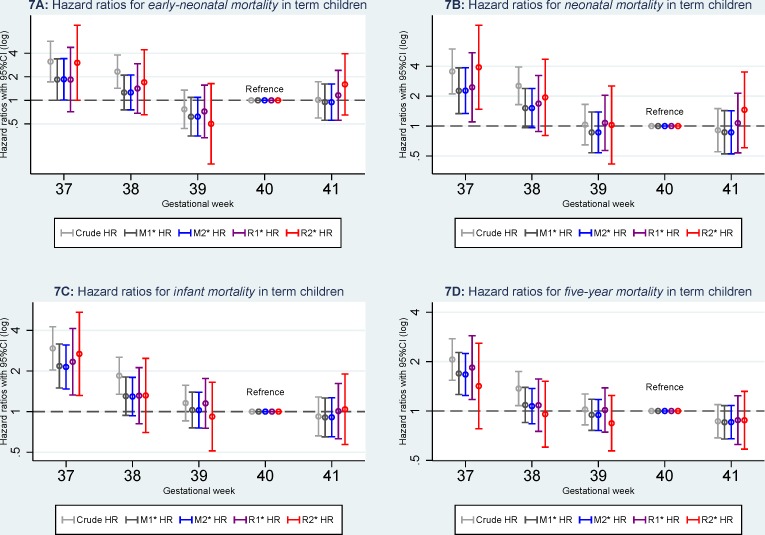
Hazard ratios for mortality according to gestational age in weeks. M1: Model 1, the results were adjusted for caesarean section (yes or no), maternal age (<25, 25–30, 30–35, > = 35), parity (1, 2, and 3), and birth year (1997–1999, 2000–2002, 2003–2005, and 2006–2008). M2: model 2, the results were additionally adjusted for maternal education (low, middle, and high) and maternal cohabitant status during birth (yes, no). R1: Restricted analyses 1, the analyses were restricted to pregnancies without registered morbidity within one year before delivery or within 30 days after delivery using the same adjustments in Model 2. R2: Restricted analyses 2: the analyses were further restricted to pregnancies without registered morbidity within one year before delivery or within 30 days after delivery and children without any registered congenital malformations using the same adjustments in Model 2.

When gestational age in days was categorized as week, an increased mortality remained higher (Fig 7) for children born week 37 for *early-neonatal mortality* (HR3.02, 95%CI: 1.00–9.09), *neonatal mortality* (HR = 3.91, 95%CI: 1.48–10.33), and *infant mortality* (HR = 2.68, 95%CI: 1.31–5.45) even excluding children born with any congenital malformations but not for *five-year mortality* (HR = 1.42, 95%CI: 0.78–2.59).

**Fig 8:**
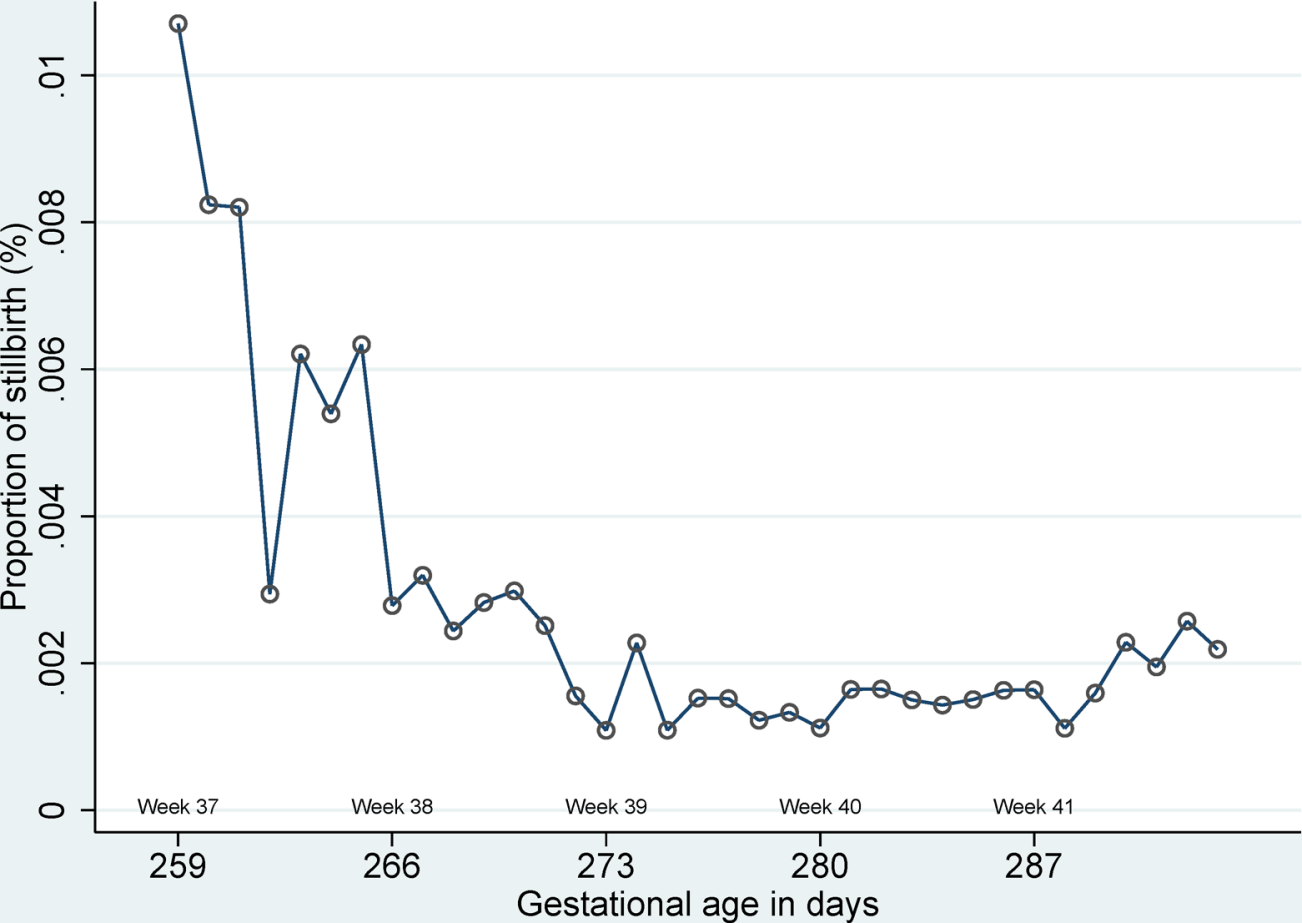
Proportion of stillbirth according to gestational age in days.

Information on gestational age was obtained for 1,527 stillbirth out of a total of 1,549 stillbirths (98.6%) from 1997 to 2004. A total of 720 (47.2%) of term stillbirths took place between 37 and 41 of gestational week and 11 out of 720 stillbirth only had information on completed gestational week but missing data on gestational days.). The highest proportion of stillbirth was observed at 37 of gestational age (Fig 8).

## Discussion

The highest risk of death was observed for children born at week 37 of gestational week. Both short-term and long-term mortality decreased with increasing gestational age in days.

When gestational age in days was classified as gestational week, the mortality for children born at 37-gestataion was higher compared to later-term birth[4,6,18,19] but we did not observe an increased mortality for children born at 38-gestataion. Therefore, classifying both 37 and 38 weeks of gestation as early-term will hide the true underlying mortality for children born in week 37.

A spontaneous childbirth before its optimal due date has reasons it could be diseases in the mother or the child to explain the increased child mortality.[20] The attenuation of mortality when pregnancies with any morbidity before and during pregnancy were excluded shown as expected that maternal morbidity affect mortality in the offspring. However, short term (including early-neonatal, neonatal, and infant) mortality remained higher but not for long-term (five-year) morality after excluding pregnancies with any registered morbidity within one year before delivery or within 30 days after delivery and excluding births with any congenital malformations, which could indicate that the timing of birth is a risk factor in itself and it is independently associated with at least short term mortality. Therefore, using the label ‘early term’ for babies being born in 37 week may give a most justified message.

We had almost complete follow-up and selection bias does not explain the results. The trend of stillbirth also decreased with increasing gestational age and highest risk of stillbirth was also observed at 37 of gestational week. We could adjust for several potential confounders but not for maternal smoking, maternal body mass index, but restricting analyses among pregnancies without any comorbidity should reflect true mortality of children born to healthy or health-conscious pregnancies.

Pregnancies with parity equal or bigger than four may be different in many ways compared to pregnancies with parities smaller than four. In order to disentangle when gestational age is independently associated with mortality, we excluded any pregnancies with parity equal or bigger than four, therefore, the findings may not necessarily reflect this population.

## Conclusions

This study shows heterogeneity in mortality rates among singletons born at term according to gestational age in days. The highest mortality and stillbirth was observed in children born at 37 weeks of gestation, therefore, 37 weeks of gestational age should be categorized as early term birth. Gestational age is independently associated with short term mortality but longer follow-up is needed to observer if gestational age is independently associated with long-term mortality.
